# Asymmetry in personal space and distance regulation during one-on-one situations in ball sports

**DOI:** 10.3389/fspor.2026.1815068

**Published:** 2026-04-13

**Authors:** Kyosuke Oku, Hirosuke Oku, Noriyuki Kida

**Affiliations:** 1 Faculty of Arts and Sciences, Kyoto Institute of Technology, Kyoto, Japan; 2 Tropical Biosphere Research Center, University of the Ryukyus, Okinawa, Japan

**Keywords:** ball games, distance perception/physiology, distance regulation, one-on-one, personal space (PS), skill level, soccer (football)

## Abstract

Subtle differences in interpersonal distance often determine performance success or failure in sports. While these distances are perceived differently depending on the game situation and relationship with the opposing player, little is known about how athletes perceive them. In this study, we first investigated whether the same distance is perceived differently using the psychological concept of personal space. We further examined how these differing perceptions affect distance regulation in ball games such as soccer. In ball games, personal space is defined as whether they felt “the ball would be taken away” during offense, or “they would be passed” during defense. In Experiment 1, participants rated how uncomfortable they felt facing an opponent during offensive and defensive situations at 10 points separated by 50-cm intervals along a 5-m line. In offensive situations, being approached by an opponent increases discomfort, because it increases the risk of losing the ball. Conversely, in defensive situations, shorter interpersonal distances reduce discomfort, as they increase the chance of taking the ball. Experiment 2 examined the initial distance of discomfort when facing an opponent with a higher skill level. On perceiving the threat of better-skilled opponents, participants kept a greater distance during offense whilst keeping shorter distance during the defense to overcome discomfort. Taken together, these findings reveal offense–defense asymmetry in distance perception and regulation. This study provides the first quantitative evidence that personal space in sports is context-dependent and strategically modulated. Our findings may be helpful for more concrete and appropriate coaching regarding distance perceptions in educational and sporting contexts.

## Introduction

In sports, a slight difference in distance can lead to victory or defeat. For example, in a one-on-one situation in a ball game, the offense moves to break through, while the defense moves to steal the ball, both constantly adjusting their distance to gain an advantage at that moment. This interpersonal distance has been studied under in “proxemics.” Proxemics comprises two concepts: “interpersonal distance” and “personal space” (1). Previous research examining the temporal dynamics of interpersonal distance found that a 2.8-m boundary marks a shift from a synchronous phase in kendo, where both players move closer or farther apart, to an asynchronous phase, where one approaches while the other retreats (2). While such research suggests that strategies may vary as physical distance changes, research on how interpersonal distance is perceived in one-on-one situations remains limited.

Personal space however refers to the distance at which a person feels uncomfortable when approached by others. The concept of human personal space was first proposed by Sommer (3), who discovered that individuals with schizophrenia experience discomfort at greater distances than healthy individuals (3). Hall extended Sommer's line of thinking and defined personal space as a 45–120 cm zone of interpersonal distance (4). The commonly used definition states that it is the area around an individual that cannot be entered without causing discomfort (5). This uncomfortable distance decreases when the person facing it is perceived as an ally (6) and is strongly influenced by the relationship between the individuals involved.

Despite its usefulness as a method for examining distance perception, the application of the psychological concept of personal space has been limited to sports contexts. Previous research applying personal space to sports found that boxers were more tolerant of being approached in daily life than social dancers (7). However, previous studies have not examined distance perception in game situations. In particular, studies are limited to examining personal space in team sports such as soccer, in contrast to one-on-one sports.

Peripersonal space is a concept that is closely related to personal space. The peripersonal space was discovered because of the presence of frontoparietal neurons that react to objects near the body (8, 9). The space to which these neurons respond is termed the peripersonal space (10, 11), which protects the self from approaching objects (12, 13). In this peripersonal space, the reactive space expands against objects recognized as harmful, such as bees (14). Furthermore, recalling situations where one can control others’ actions, such as through social power, makes people more tolerant of others approaching them (15). Thus, the evaluation of an object by an individual is considered crucial in defining the peripersonal space.

Previous research on distance perception has shown that people's perception of distance can vary depending on the situation, and whether or not they perceive the other person as a threat. Applying this psychological concept to sports contexts leads to the hypothesis that distance perception differs between offensive and defensive situations (Experiment 1). We further hypothesized that such perceptual differences influence players’ distance regulation strategies, particularly when facing higher-skilled opponents as threats (Experiment 2). Both experiments were conducted using the personal space method, which is well-established in the field of psychology. This study focused on ball sports soccer because the offensive and defensive roles are clear, and the large court size allows for a detailed analysis of distance perception. This study provides a quantitative framework for evaluating distance perception in sports based on the psychological concept of personal space. It is expected to contribute to more specific and effective coaching in education and athletic instruction.

## Materials and methods

This study was conducted in accordance with the Declaration of Helsinki and approved by the Research Ethics Committee of Kyoto Institute of Technology. Written consent was obtained from all participants after explaining the purpose of the study.

### Experiment 1

#### Participants

The experiment was conducted with 18 male university soccer team members (age [mean ± standard deviation: 19.69 ± 1.51 years). Only male participants were included to reduce variability in perceived interpersonal distance related to sex differences in sport performance and physical characteristics. Previous studies have suggested that spatial perception and sport performance may differ between males and females (1, 16, 17). Therefore, the present study focused on a homogeneous male sample to examine the basic characteristics of distance perception in soccer. The sample size was determined based on prior research in the field of motor cognition (18–20). To ensure comparable skill levels, a team of approximately 160 players was divided into five categories and participants were selected from the same category.

#### Procedure

**Figure 1:**
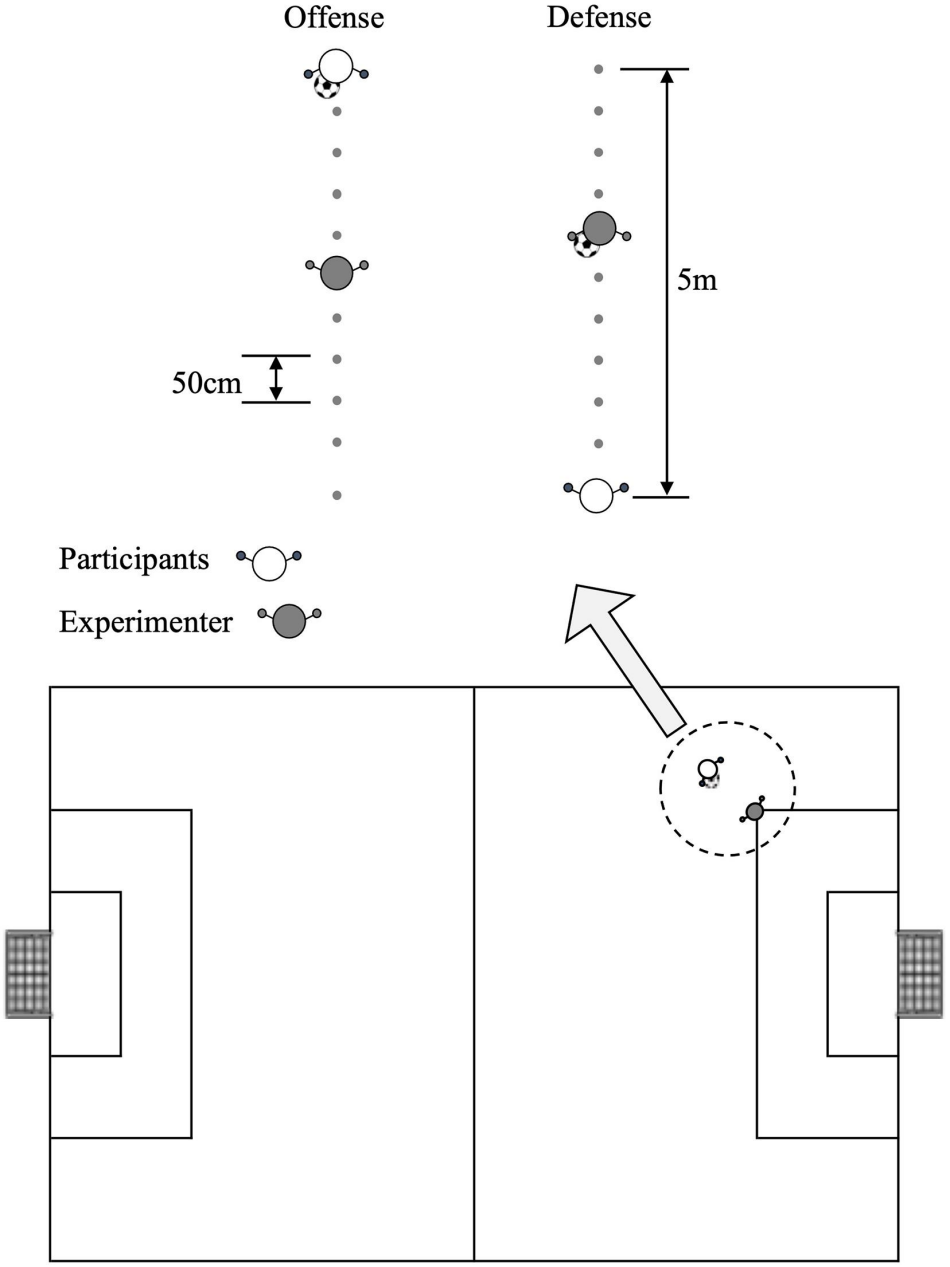
**Experimental setup for the magnitude method.** The 5 m distance from the participant was divided into 50 cm intervals. Participants rated discomfort levels as an experimenter who moved randomly along these positions. In the case of offensive situations, participants held the ball, while defensive situations, the experimenter held it. Measurements were taken at the actual court positions shown in the lower panel, which simulated a one-on-one situation near the sideline.

We adopted the magnitude method, an established technique for measuring personal space based on prior research (18, 21, 22). This method involves participants reporting their level of discomfort at each distance to an experimenter standing randomly at various distances. Participants were asked to rate on a 10-point scale (1–10) whether they feel “the ball might be taken away” during offense or “they might be beaten” during defense. The assumed scenario for the players was “a one-on-one situation near the sideline created by a short pass from the center or the same side, where the attacker had not yet gained sufficient speed.” Ten flat markers were placed at 50 cm intervals along a 5-m line in front of the participant's position (Figure 1). For offensive personal space, participants held the ball while the experimenters randomly moved along a 5-m line. Participants rated discomfort at each marker position, “feeling the ball might be taken away” was rated 10, while “not feeling the ball might be taken away” was rated 1. For the defensive personal space, the experimenter held the ball, moved it randomly along the line, and rated the discomfort at each position, as mentioned above. The measurements were conducted at locations that replicated the situation in an actual court (Figure 1). After the measurement, the participants provided free-response explanations as to why they felt discomfort.

### Experiment 2

#### Participants

A questionnaire was administered to 119 male university soccer team members [age (mean ± standard deviation): 19.82 ± 1.28 years]. The sample size was determined by referencing previous questionnaire-based studies (23). Only male participants were included to maintain consistency with Experiment 1 and to reduce variability associated with sex differences in physical ability and sport performance (18, 21, 22).

#### Procedure

**Figure 2:**
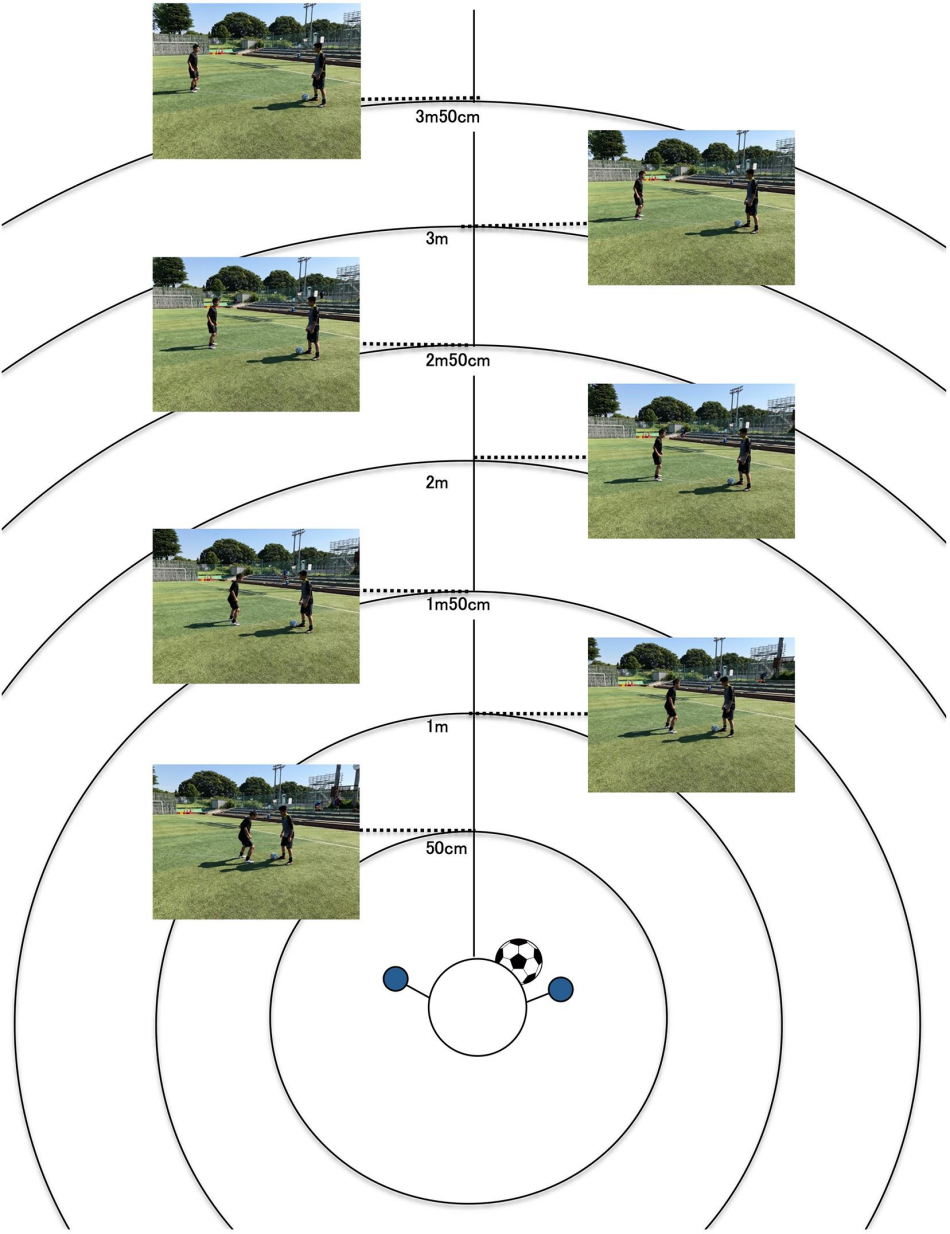
**Questionnaire used to measure personal space.** Participants were asked to place stickers at the distance where they “began to feel the ball might be taken away” during offensive situations and at the distance where they “began to feel they might be beaten” during defensive situations.

Based on established questionnaire methods for measuring personal space (24), we measured personal space in soccer by defining a boundary at the distance at which discomfort begins when an opposing player approaches. The questionnaire method was selected because it enabled participants to imagine opponents with higher skill levels. A questionnaire (Figure 2) was created to measure personal space in relation to opponents’ skill levels. Participants were asked to indicate the distance at which discomfort began in four scenarios: offensive vs. defensive situations  ×  perception of opponents’ skill levels (superior vs. equal to the self). In offensive situations, the participants placed stickers at a distance where they began to feel that the ball might have been taken away. In defensive situations, stickers are placed at a distance where they begin to feel that they may be beaten. The stickers were circular with a diameter of 20 mm and placed to represent the opponent. Questionnaires were distributed and collected through group surveys. The 50 cm intervals on the illustrated questionnaire (Figure 2) were created on a 2.5 cm (1/40 scale) scale to enable the calculation of actual distances. Third-person perspective photos captured at actual distances were attached to aid in the perception of distance. The assumed game situation was identical to that in Experiment 1: “A one-on-one situation near the sideline created by a short pass from the center or same side, where the attacker has not yet fully accelerated.”

## Results

### Experiment 1

**Figure 3:**
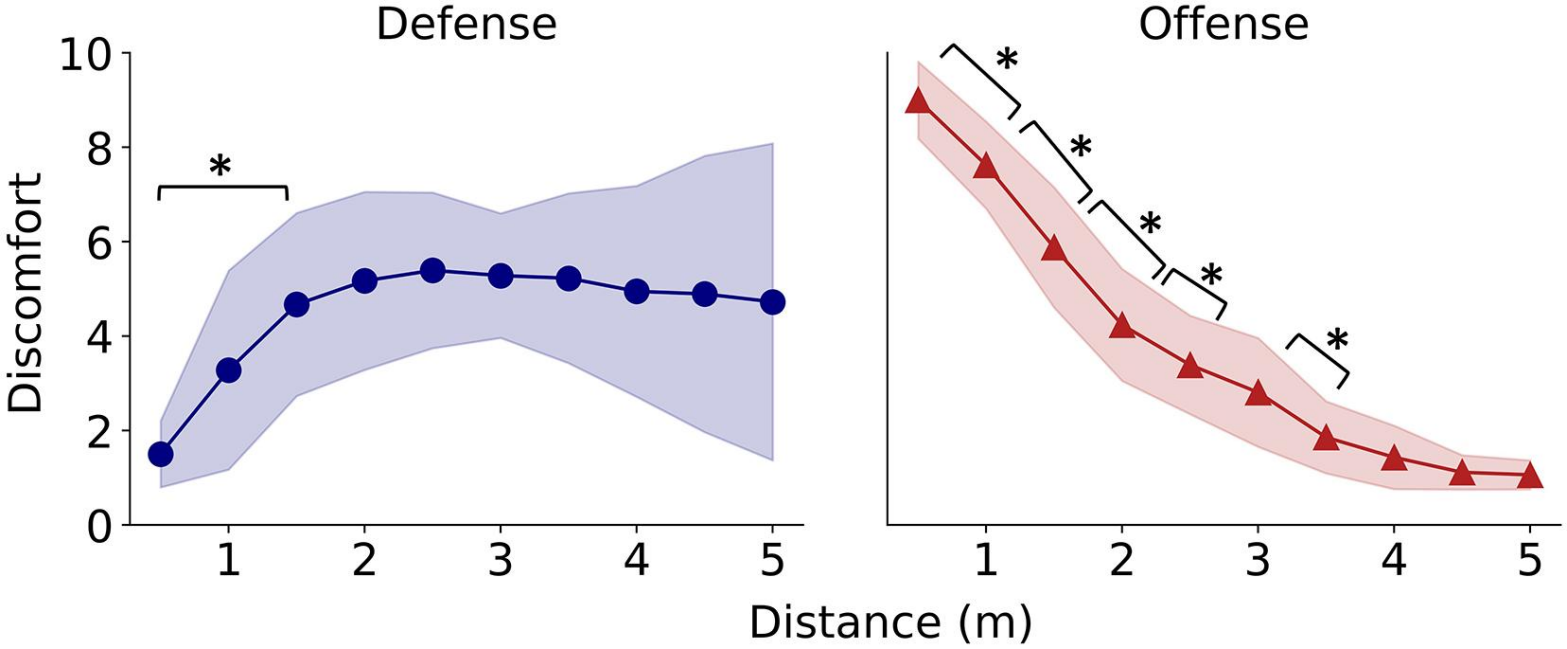
**Changes in discomfort with distance during defense (left panel) and offense (right panel).** The colored bands represent the standard deviation. A single asterisk indicates significance at *p* < 0.05.

As shown in Figure 3 left panel, participant discomfort increased with increasing distance and likely reached a plateau beyond 2 m during the defense phase. In contrast, discomfort was maximal at the closest opponent's position (50 cm) and sharply decreased as the distance increased (Figure 3, right panel).

The results of statistical analysis for the defensive situation are shown in Figure 3 (left panel). A one-way analysis of variance (ANOVA) confirmed a main effect of distance [*F* (9,153) = 6.16, *p* < 0.001, *η^2^* = 0.23]. Multiple comparisons revealed significant differences in discomfort between the closest point (50 cm) and all distances farther than 1.5 m (*p* < 0.05).

The results of the statistical analysis for the offensive situations are presented in Figure 3 (right panel). A one-way ANOVA confirmed the main effect of distance [*F* (9,153) = 273.18, *p* < 0.001, *η^2^* = 0.90]. Multiple comparisons revealed significant differences between adjacent distances: 50 cm and 1 m, 1 m and 1.5 m; 1.5 m and 2 m, 2 m and 2.5 m; and 3 m and 3.5 m (*p* < 0.05).

### Experiment 2

**Figure 4:**
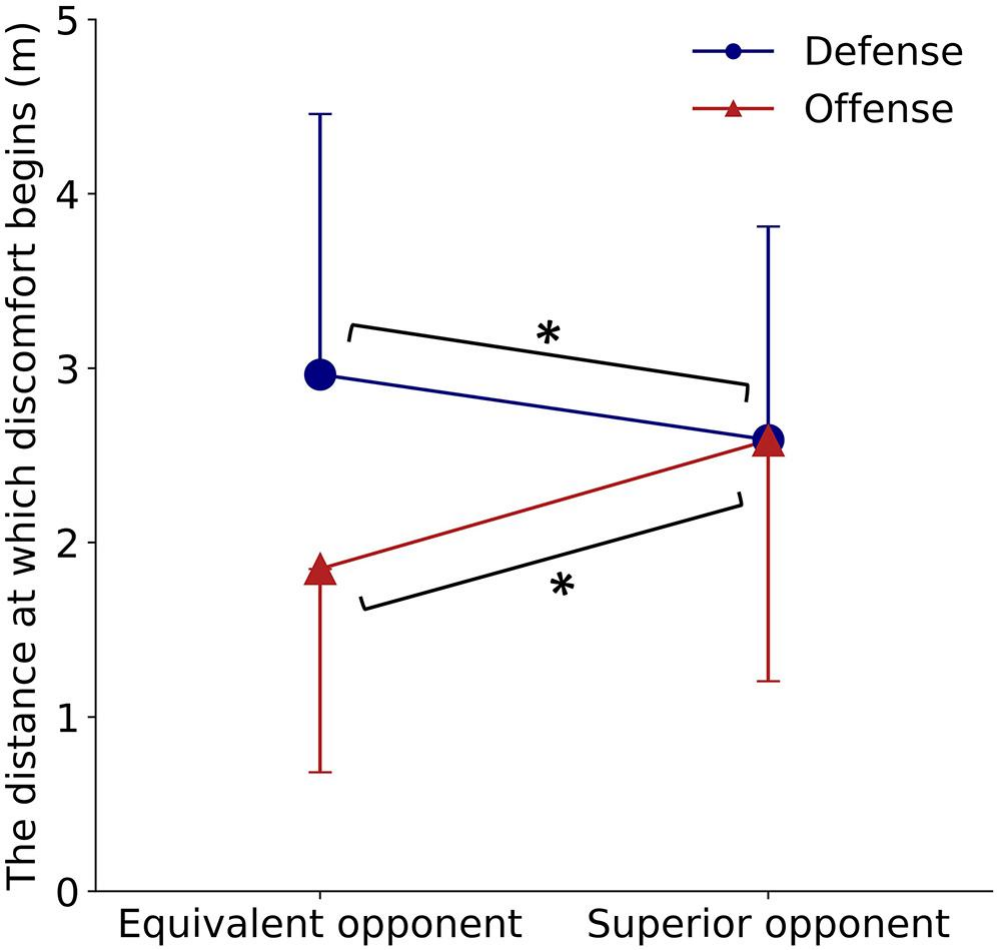
**Changes in personal space according to game situation and opponent skill level.** The whiskers represent the standard deviation. A single asterisk indicates significance at *p* < 0.05.

A two-way ANOVA was conducted on personal space, with the game situation (offense/defense) and perception of the opponent's skill level (superior/equal) as within-subject factors (Figure 4). Although the effect size was small, a significant interaction was observed [*F* (1,118) = 57.33, *p* < 0.001, *η^2^* = 0.04]. Similarly, significant main effects were observed for both the situation factor [*F* (1,118) = 22.95, *p* < 0.001, *η^2^* = 0.04] and the perception of the opponent skill level [*F* (1,118) = 5.66, *p* = 0.019, *η^2^* = 0.004], despite the small effect size. Multiple comparisons revealed that players tended to approach more closely when defending against opponents with a superior skill level [2.58 ± 1.22 m, 95%CI: (2.37, 2.81)] than when facing opponents of an equal skill level [2.96 ± 1.49 m, 95%CI: (2.69, 3.24)] (*p* < 0.05). Conversely, in an offensive situation, players tended to maintain a larger distance when facing opponents with a higher skill level [2.59 ± 1.38 m, 95%CI: (2.34, 2.84)] than when facing opponents of an equal skill level [1.85 ± 1.17 m, 95%CI: (1.64, 2.06)] (*p* < 0.05).

## Discussion

This study aims to investigate distance perception in ball sports using the psychological concept of personal space, and to examine how these perceptions affect playing strategy. We hypothesized that the same distance could be perceived differently depending on the situation (offense vs. defense), which would affect distance regulation. Our results supported this hypothesis, demonstrating that the asymmetry of personal space in ball sports influences distance regulation.

In Experiment 1, participant discomfort increased with distance and reached a plateau beyond 2 m during the defense phase. The discomfort response for the offensive phase was completely different from the defense phase; discomfort was maximal at the closest opponent position and decreased with distance (Figure 3). Even with the same distance between opponents, participant discomfort varies depending on the game situation. Next, we examined how these distance perceptions affect playing strategy. Experiment 2 revealed that when the opponent's skill level was higher than one's own, players tended to try to keep them farther away during offensive situations and closer during defensive situations (Figure 4).

The results of Experiments 1 and 2 can be summarized in each situation: in an offense situation, discomfort increases as the opponent approaches (Experiment 1), so players adopt a strategy to keep them farther away when the opponent's skill level is higher (Experiment 2). In a defense situation, discomfort decreases as the opponent approaches (Experiment 1), leading to a strategy of moving closer when facing higher-level opponents (Experiment 2). These findings suggest that the perception of the same distance differs between offensive and defensive situations, followed by changes in strategies adapted according to the opponent's skill level and perceived threat.

Notably, the results of Experiment 1 for the offense phase partially agree with the results of psychological research on personal space. In psychology, there is general agreement that personal space is characterized by increasing discomfort as proximity increases (1). Furthermore, research on the peripersonal space indicates that when an approaching object is perceived as harmful, there is a tendency to push it further away (14). The offensive results from Experiments 1 and 2 in this study are in good agreement with these characteristics, where discomfort increases with proximity and harmful objects are pushed away. These findings demonstrate that the psychological concept of personal space can be applied to sports.

In contrast, discomfort decreased as the distance decreased during defense. This observation cannot be explained by psychological personal-space theory. To clarify why the discomfort decreased, a questionnaire was administered to the participants in Experiment 1. The answers to this questionnaire disclosed that players thought “shortening the distance allows them to steal the ball” during defense situation. This suggests that confidence in one's ability to steal the ball overrides the perceived threat of being beaten during defense. Combining these results with those from Experiment 1, the 1.5 m range where discomfort significantly decreases (Figure 3, left panel) is likely the threshold where players perceive that they can win the ball. This change in discomfort has not been explained by previous personal space research in psychology and was confirmed in sports for the first time.

A concept closely related to personal space is affordance. Affordances are defined as possibilities provided by the environment relative to an individual's capabilities (25). Previous research has shown that affordances influence how interpersonal space is perceived (17, 26–28). The discomfort measured in the present study may reflect the perception of affordances in the given situation. In defensive contexts, for example, approaching the opponent may simultaneously increase the affordance of stealing the ball while decreasing the affordance for the opponent to advance. In this sense, the reported discomfort may represent the subjective experience accompanying these perceived action possibilities.

In ball sports, the affordances available to players can depend on the spatial configuration and roles of surrounding players. Previous research has shown that affordances in soccer can vary depending on whether surrounding players are teammates or opponents, as well as their body orientation (29). However, the present study focused on a one-on-one situations involving a single opponent. Therefore, variations in affordances created by multiple players (e.g., teammates and opponents forming passing gaps) were not directly manipulated in the present design.

Furthermore, players adopt the strategy of getting closer to threatening opponents to reduce discomfort during defense. Many people are likely to experience tension when faced with skilled opponents in sports. According to the Yerkes-Dodson law, anxiety and performance follow a U-shaped curve, whereby excessive anxiety impairs performance (30). Numerous sports studies have reported that high anxiety levels tend to decrease performance (31, 32). Most approaches, such as mental training to manage tension or discomfort (33), are commonly used outside of real-time gaming situations. Therefore, this study is the first to reveal how personal space changes based on the game situation, providing more insight into strategies that can improve performance.

The findings of this study are also applicable to other ball sports. In ball sports, the player with the ball is usually offensive and the player without the ball is defensive, which makes the player's situation clear. In contrast, in sports such as boxing (7) or kendo (2), offense and defense occur constantly, and the offense and defense phases change instantly. Furthermore, the distance between players is very small compared to that in ball sports. Therefore, defining discomfort in such sports is difficult. However, the results of this study can be generalized to ball sports, where offense and defense are clearly defined, and personal space can be more easily defined according to each situation. This means this research is highly applicable to other ball sports.

It should also be emphasized that the simple measurement method adopted in this study can be easily applied to other sports. The magnitude method used in Experiment 1 only required participants to stand and report their feelings about the distance, allowing the experiment to be completed in approximately 5 min. The questionnaire used in Experiment 2 can be administered indoors, even for sports that are usually played outside. Furthermore, the Experiment 2 questionnaire included photographs illustrating each distance to aid the participants’ visualization. Previous research has confirmed that there is no difference between first- and third-person perspectives in virtual personal space measurements (1). This ensured the validity of the questionnaire results used in this study. These measurement methods enable the simple quantification of ambiguously presented distance perceptions and facilitate more concrete coaching in education and athletic instruction, that is, determining how close a defender can approach an attacker.

## Limitations

We cannot say with certainty how the perceptions of distance vary in dynamic situations. Experiments 1 and 2 assume static scenarios. However, in sports, players must make decisions while adapting to constantly changing environments. Even for the same distance, the perception may differ depending on whether the distance is closing or moving away. Future research should use VR to recreate dynamic situations, thereby enabling a more detailed investigation of distance perception in sports.

Another limitation is that the body orientation was not manipulated. Previous research has shown that the perceived width of passing lanes can vary depending on the body orientation of surrounding players (29). For example, gaps created by opponents facing toward the passing lane are perceived as narrower than those created by players facing away. Similarly, the perceived threat from an opponent in a one-on-one situation may vary depending on both the opponent's body orientation and the player's own orientation. Future research should investigate how these factors influence perceived discomfort and distance regulation.

A final limitation is that all participants in this study were male soccer players. Sex differences have been reported in sport performance and spatial perception (18, 21, 22), which means that the present findings cannot be directly generalized to female athletes. Future research should include female participants to examine whether the offense–defense asymmetry in distance perception is consistent across sexes.

## Conclusion

This study aimed to investigate whether the perception of the same distance varies depending on the situation in ball sports such as soccer and how these perceptual differences influence playing strategies. Experiment 1 suggested that the perception of the same distance differed between offense and defense: players felt more vulnerable to losing the ball when approaching during offense and more secure against being dribbled when approaching during defense. Experiment 2 revealed that when facing opponents of higher skill levels, players tended to try to keep opponents at a greater distance during offense and at a closer distance during defense. The results from Experiments 1 and 2 suggest that distance perception differs between offense and defense in one-on-one soccer situations, and that this difference in perception may influence distance regulation. This study is the first to apply the concept of personal space to ball sports and to clarify the previously ambiguous concept of distance perception. Our study will enable more specific instruction in education and athletic coaching.

## Data Availability

The original contributions presented in the study are included in the article/Supplementary Material, further inquiries can be directed to the corresponding author.
